# Manipulating insulin signaling to enhance mosquito reproduction

**DOI:** 10.1186/1472-6793-9-15

**Published:** 2009-08-20

**Authors:** Anam J Arik, Jason L Rasgon, Kendra M Quicke, Michael A Riehle

**Affiliations:** 1 Department of Entomology – University of Arizona, Tucson, AZ, USA; 2 The W. Harry Feinstone Department of Molecular Microbiology and Immunology – Bloomberg School of Public Health, Johns Hopkins University, Baltimore, MD, USA; 3 The Johns Hopkins Malaria Research Institute – Johns Hopkins University, Baltimore, MD, USA

## Abstract

**Backgrond:**

In the mosquito *Aedes aegypti *the insulin/insulin growth factor I signaling (IIS) cascade is a key regulator of many physiological processes, including reproduction. Two important reproductive events, steroidogenesis in the ovary and yolk synthesis in the fat body, are regulated by the IIS cascade in mosquitoes. The signaling molecule phosphatase and tensin homolog (PTEN) is a key inhibitor of the IIS cascade that helps modulate the activity of the IIS cascade. In *Ae. aegypti*, six unique splice variants of AaegPTEN were previously identified, but the role of these splice variants, particularly AaegPTEN3 and 6, were unknown.

**Results:**

Knockdown of AaegPTEN or its specific splice variant AaegPTEN6 (the splice variant thought to regulate reproduction in the ovary and fat body) using RNAi led to a 15–63% increase in egg production with no adverse effects on egg viability during the first reproductive cycle. Knockdown of AaegPTEN3, expressed predominantly in the head, had no effect on reproduction. We also characterized the protein expression patterns of these two splice variants during development and in various tissues during a reproductive cycle.

**Conclusion:**

Previous studies in a range of organisms, including *Drosophila melanogaster *and *Caenorhabditis elegans*, have demonstrated that disruption of the IIS cascade leads to decreased reproduction or sterility. In this study we demonstrate that knockdown of the IIS inhibitor PTEN can actually increase reproduction in the mosquito, at least during the first reproductive cycle.

## Background

Mosquito-borne diseases such as dengue, malaria and lymphatic filariasis are an increasing global health problem. Dengue, along with dengue hemorrhagic fever (DHF), is transmitted by the mosquito *Aedes aegypti *and is becoming an increasing threat in more than one hundred countries [1]. A better understanding of the mosquito's reproductive physiology could lead to novel control strategies that could complement or replace current methods of control. It has been theorized that increased reproductive effort results in a trade-off with lifespan. Such a trade-off is supported by studies conducted on a wide range of organisms, including birds, mammals, fruit flies and roundworms [2-4]. However, recent studies also indicate that lifespan and reproduction can be uncoupled [5]. The insulin/insulin growth factor I signaling (IIS) cascade lies at the heart of this interplay between reproduction and lifespan in eukaryotic organisms [6-8]. Studies in *Caenorhabditis elegans *and *Drosophila melanogaster *show that gene mutations in signaling molecules within the IIS cascade affect lifespan and reproduction [9-12]. A well conserved IIS cascade has also been identified in mosquitoes and, as in other organisms, it plays an important role in regulating lifespan and reproduction [8,13,14]. In the yellow fever mosquito, *Aedes aegypti*, the IIS cascade controls the production of ecdysteroids in the follicle cells surrounding the ovary [8]. These ovarian ecdysteroids regulate yolk production in the fat body and egg development. The IIS cascade has also been implicated in the process of vitellogenesis itself, where insulin signaling is one of the components regulating the gene expression of yolk protein precursor (YPP) [15].

A key negative regulator of IIS cascade is phosphatase and tensin homolog (PTEN) that resets the cascade to its resting state [16]. Being an inhibitor of the IIS cascade, PTEN is an important candidate for controlling reproduction and lifespan at the molecular level. Originally identified as a tumor suppressor, PTEN was later found to down regulate the IIS by acting as a direct antagonist of phosphoinositide 3-kinase (PI3K) [17]. PTEN antagonizes PI3K by hydrolyzing the PI 3-phosphates at the membrane, negatively regulating the insulin signaling pathway and its various downstream events. In mammals, PTEN has been shown to control ovarian follicle activation and hence reproduction [18]. In mice, PTEN is expressed in a cell specific manner in the ovary. Mutant mice with suppressed PTEN are fertile, ovulate more oocytes and produce moderately more pups than control mice [19]. The *Drosophila *orthologue of PTEN, dPTEN, reduces the sizes of follicle cells following an increase in cell size due to high dAkt^myr ^expression [20].

Multiple splice variants of PTEN have been identified in various organisms. In humans, differential expression of three PTEN splice variants has been predicted to influence different phenotypes [21]. Three splice variants of the *Drosophila *orthologue of PTEN (dPTEN) have been identified and all three are expressed throughout development as active phosphatases [22]. In *Ae. aegypti*, six splice variants of AaegPTEN were identified [23]. These splice variants appear to be unique to mosquitoes and were shown to be differentially expressed during mosquito development and in adult tissues [23]. Three of these splice variants, AaegPTEN1, 4 and 5, arise through intron retention, resulting in the early termination of translation. These splice variants are expressed at low levels in all tissues and are likely to be quickly degraded since they lack the C-terminal regulatory sites. The other three splice variants, AaegPTEN2, 3 and 6 arise from alternative splicing of the terminal exon, which results in the expression of full length, bioactive proteins. AaegPTEN6 is the only splice variant with a PDZ binding motif. AaegPTEN2, 3 and 6 splice variants have unique expression patterns. AaegPTEN2 transcript is only expressed in the ovaries at very low levels. AaegPTEN3 transcript is expressed predominantly in the head and to a lesser extent the midgut, two key tissues involved in lifespan regulation in *D. melanogaster *and *C. elegans *[24-26]. AaegPTEN6 transcript is expressed predominantly in the fat body and ovary, key reproductive tissues in insects [15,23]. The possibility that a single splice variant (PTEN6) is involved in regulating both ovarian ecdysteroids production and yolk production in the fat body has been proposed [23].

The aim of this study was to determine if we could manipulate mosquito reproduction by manipulating the IIS cascade. Towards this goal we examined two splice variants of AaegPTEN (3 and 6) in the mosquito *Aedes aegypti *and the role they play in mosquito reproduction. These two variants were selected because they encode full length phosphatases that are highly expressed in a range of tissues. We determined the protein expression patterns of AaegPTEN3 and 6 at different stages of development and in different adult tissues. We explored the dynamics of AaegPTEN3 protein expression in the head and AaegPTEN6 in fat body, midgut and ovaries during a reproductive cycle and post oviposition. Most importantly, we knocked down expression of the AaegPTEN splice variants and demonstrated a significant increase in egg production with the AaegPTEN6 splice variant knockdown.

## Results

### Protein expression pattern of AaegPTEN3 and 6 in distinct tissues

**Figure 1:**
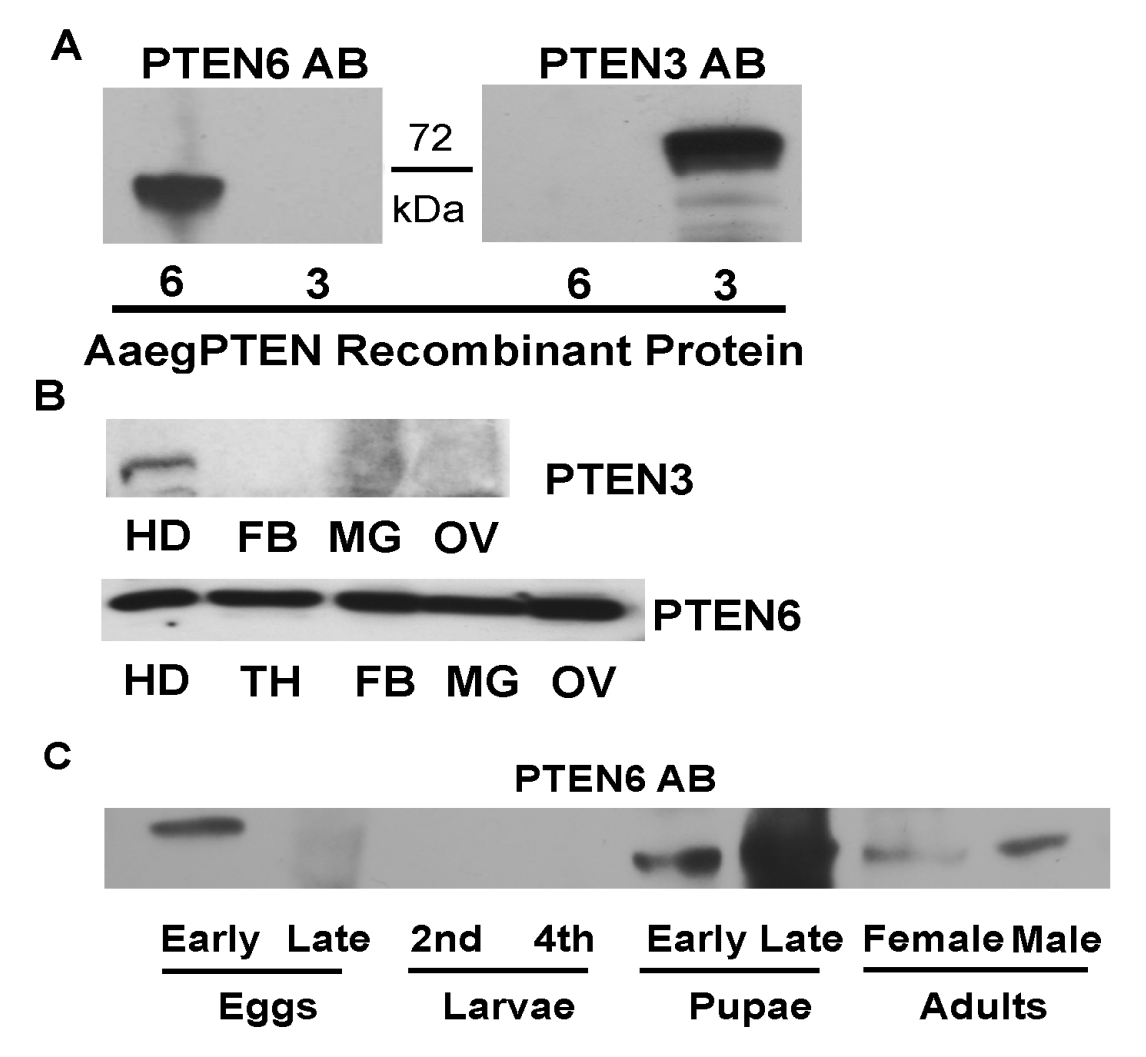
**Protein expression of AaegPTEN3 & 6 in female tissues**: AaegPTEN3 and 6 expression in various female tissues was detected using immunoblot analysis. Tissues from 3–5 day old female mosquitoes were dissected in 10× protease inhibitor (PI) buffer, size fractioned on 12% SDS-PAGE gels, transferred to PVDF membrane and probed with anti-AaegPTEN3 or 6 antibodies. **A**. AaegPTEN3 and 6 recombinant proteins were immunoblotted and each were probed with the AaegPTEN3 or 6 antibodies. Both the AaegPTEN3 and 6 antibodies were specific to the appropriate recombinant protein and did not recognize the other splice variant. **B**. AaegPTEN6 was detected in all tissues from adult females examined, including non-bloodfed heads (HD), thoraxes (TH), fat bodies (FB), midguts (MG) and ovaries (OV). AaegPTEN3 could only be detected in the head. **C**. AaegPTEN6 was expressed in early embryos, pupae and adults. However, it was found at very low levels in diapausing eggs and not at all during larval stages.

The transcript expression patterns of all six AaegPTEN splice variants have been previously characterized [23]. In this study it was shown that *AaegPTEN6 *was predominantly expressed in reproductive tissue (ie the ovary and fat body) and to a lesser extent in the thorax and midgut. In contrast, *AaegPTEN3 *was abundantly expressed in the head, and to a lesser extent the midgut. To examine protein expression patterns of AaegPTEN3 and 6 and compare them with the transcript expression data, we generated peptide-based polyclonal antibodies against the C-terminus of AaegPTEN3 and 6. The specificity of AaegPTEN3 and 6 specific antibodies was confirmed using AaegPTEN3 and 6 recombinant proteins. Both antibodies recognized the appropriate AaegPTEN recombinant protein with no cross reactivity (Figure 1a). This was particularly important for the AaegPTEN6 antibody which was generated against a peptide that shared four amino acids with all six splice variants. Next, we examined the protein expression of the two AaegPTEN splice variants on various mosquito tissues including heads, thorax, fat bodies, midguts and ovaries. AaegPTEN3 was strongly expressed in the head, but either absent or very weakly expressed in the other tissues (Figure 1b). This is consistent with transcript expression patterns where *AaegPTEN3 *was abundantly expressed in the head [23]. The AaegPTEN6 protein was expressed in all tissues examined, including the midgut (Figure 1b) in fairly equal amounts. This is in contrast with the transcript expression pattern of *AaegPTEN6 *where expression in the midgut is low and other tissues, such as the ovary and fat body, have moderate to high levels of expression [23].

During development, AaegPTEN6 had distinct protein expression patterns (Figure 1c). It was expressed in early embryos, pupae and adults. However, it was found at very low levels in developmentally arrested eggs (>48 h post-oviposition) and not found at all during larval stages. The high level of expression in early embryos is consistent with its strong transcript expression. Interestingly, AaegPTEN6 transcript is found at low levels in all the developmental stages even during the larval stages when we couldn't detect the protein.

### Protein expression pattern of AaegPTEN 6 during a reproductive cycle

**Figure 2:**
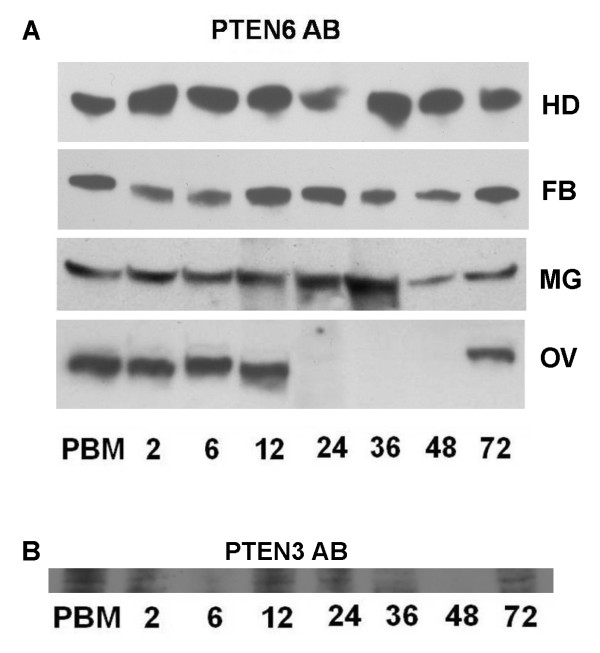
**Protein expression of AaegPTEN6 in female tissues during a reproductive cycle**: To assess the protein expression pattern during reproduction, tissue samples from fully engorged females were collected at various time points (0 (non-fed), 3, 6, 12, 24, 36, 48, and 72 hr (post oviposition)). Tissues from 10–15 females were dissected into 10× PI buffer, size fractioned on 12% SDS-PAGE gels, transferred to PVDF membrane and probed with anti-AaegPTEN 6 antibody. **A**. AaegPTEN6 was expressed at constant levels throughout the reproductive cycle in heads (HD), midguts (MG) and fat bodies (FB). However, in the ovaries (OV), AaegPTEN6 was strongly expressed prior to and during the first 12 h post-bloodmeal. It could not be detected 24 to 36 h after the bloodmeal, but was again detected at 72 h post-bloodmeal, following oviposition. **B**. AaegPTEN3 protein in the head was evenly expressed throughout the reproductive cycle. Low expression at 6 and 48 h is due to loading errors as determined by GAPDH expression (not shown).

The possibility of AaegPTEN6 being a key regulator of reproduction was indicated by the prominent expression of AaegPTEN6 transcript in two key reproductive tissues, the fat body and ovary. Before exploring this possibility we characterized the AaegPTEN6 protein expression dynamics throughout the mosquito's reproductive cycle in four key tissues: ovaries, fat bodies, heads and midguts. In ovaries, AaegPTEN6 protein was abundantly expressed during the first 12 h of the reproductive cycle and was absent in the ovaries from 24 hr post-bloodfeeding until oviposition (Figure 2a). After oviposition (72 h) the AaegPTEN6 protein reappeared in the ovaries. This pattern is similar to the expression dynamics of other insulin signaling components in the ovary e.g. Aaegp110 [27] and the mosquito insulin receptor [13]. In the fat body, head and midgut, AaegPTEN6 was expressed evenly throughout the reproductive cycle (Figure 2b). AaegPTEN3 protein in the head was also evenly expressed throughout the reproductive cycle, the very low expression at 6 and 48 h is due to a loading error (Figure 2b).

### Effects of RNAi-mediated knockdown on egg production and egg viability

**Figure 3:**
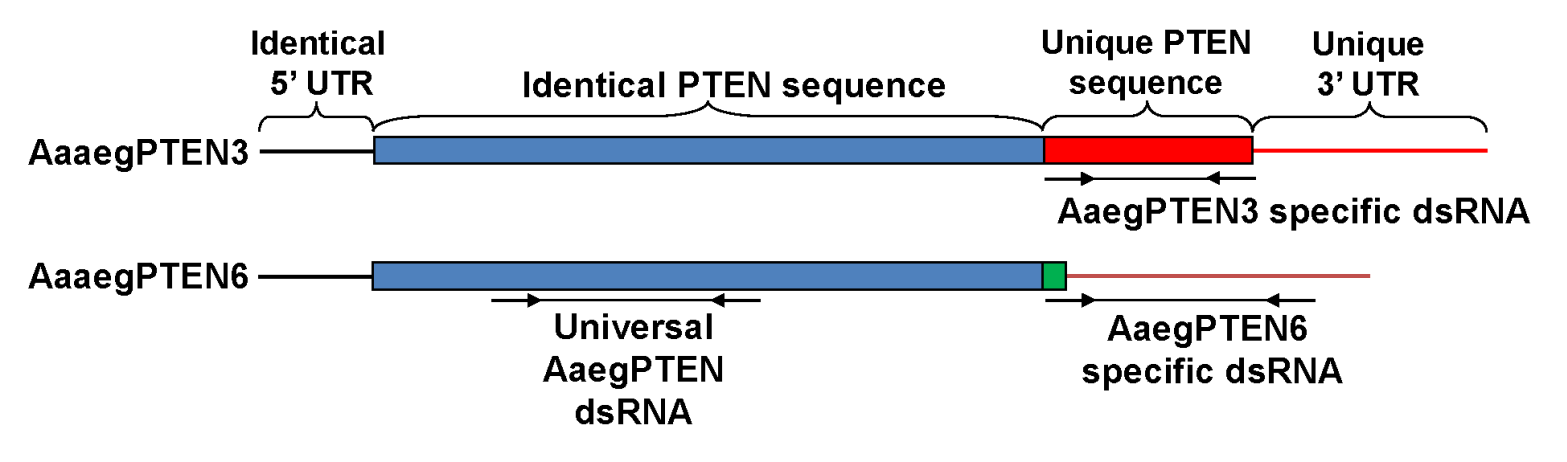
**Generation of dsRNA constructs against AaegPTEN and two key splice variants**: Three dsRNA constructs were synthesized against AaegPTEN. Universal AaegPTEN dsRNA was generated against the identical PTEN region and was capable of knocking down all AaegPTEN splice variants. The AaegPTEN3 dsRNA was generated against ~300 bp of its unique 3' terminus and should only silence the AaegPTEN3 splice variant. AaegPTEN6 dsRNA was generated against the unique 18 bp coding sequence and ~300 bp of the 3' UTR and should only silence the AaegPTEN6 splice variant. dsRNA was also generated against ~300 bp of DsRed as a control (not shown).

The effects of AaegPTEN and its splice variants on the reproductive success of female mosquitoes were examined using RNA interference. Double stranded RNA (dsRNA) was generated that specifically targeted the AaegPTEN3 and 6 splice variants. In addition, a "universal" AaegPTEN dsRNA was generated that targeted all six splice variants (Figure 3). Adult female mosquitoes (12–24 h post eclosion) were injected with 2 μg of dsRNA against AaegPTEN, its splice variants or a non-specific control dsRNA against the red fluorescent protein from *Discosoma *(DsRed). Survivorship of mosquitoes injected with dsRNA was >95% in all treatment groups and comparable to uninjected mosquitoes (data not shown).

**Figure 4:**
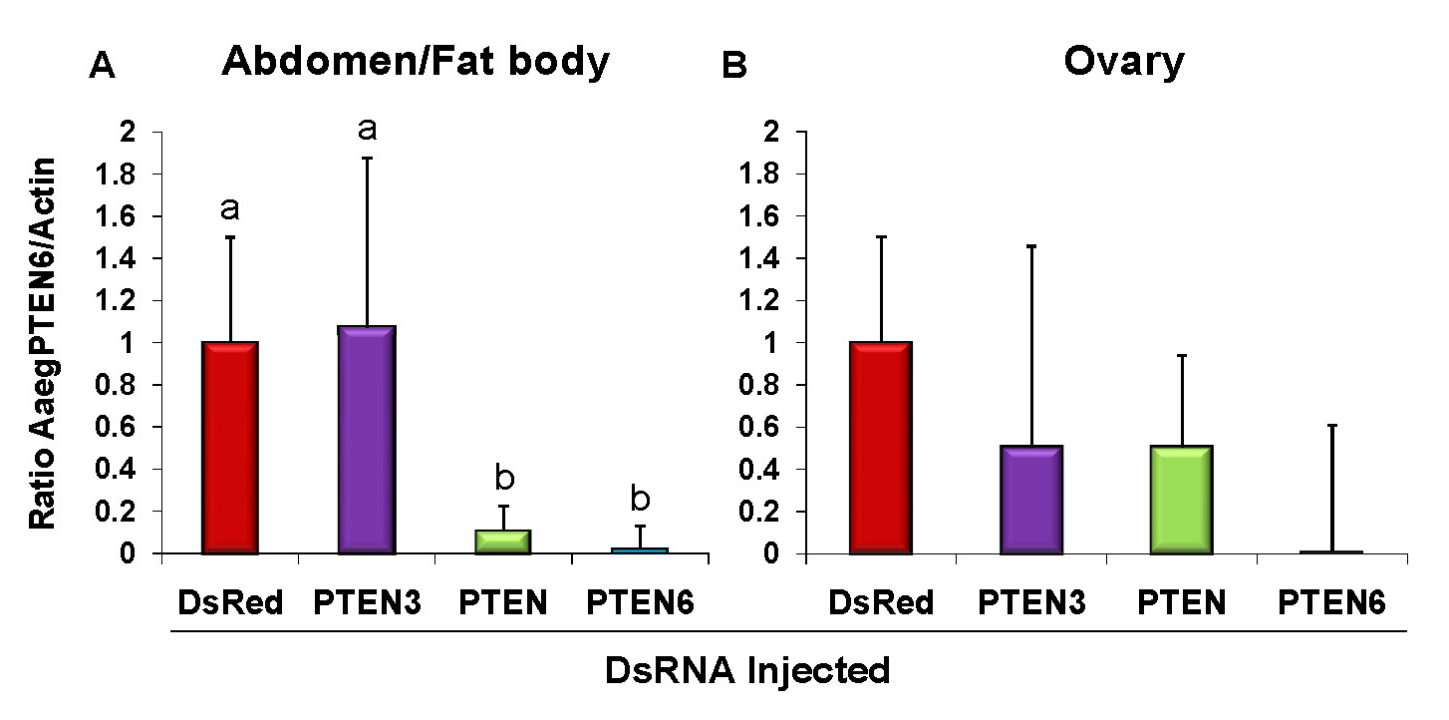
**Knockdown of AaegPTEN6 in the abdomen/fat body and ovary**: Female mosquitoes (48 h post eclosion) were injected with 2 μg of dsRNA (0.5 μl of a 4 μg/μl solution). Injected mosquitoes were allowed to recover for 48 h before being mated with male mosquitoes and provided with a bloodmeal. At 48 h post-bloodmeal (96 h post-injection) we generated cDNA from the total RNA of abdomens/fat body and ovaries to assess the transcript knockdown after specific dsRNA injections. qRT-PCR was performed using primers specific to AaegPTEN6 and actin as a control. Abdomens/fat body injected with universal AaegPTEN dsRNA had a 90% reduction in AaegPTEN6 transcript, while those injected with AaegPTEN6 specific dsRNA had a 98% reduction in AaegPTEN6 transcript. Similarily, ovaries injected with universal AaegPTEN dsRNA had a 51% reduction in AaegPTEN6 transcript, while those injected with AaegPTEN6 specific dsRNA had a 99% reduction in AaegPTEN6 transcript relative to control mosquitoes injected with DsRed dsRNA. Different letters indicate a significant difference between samples.

**Figure 5:**
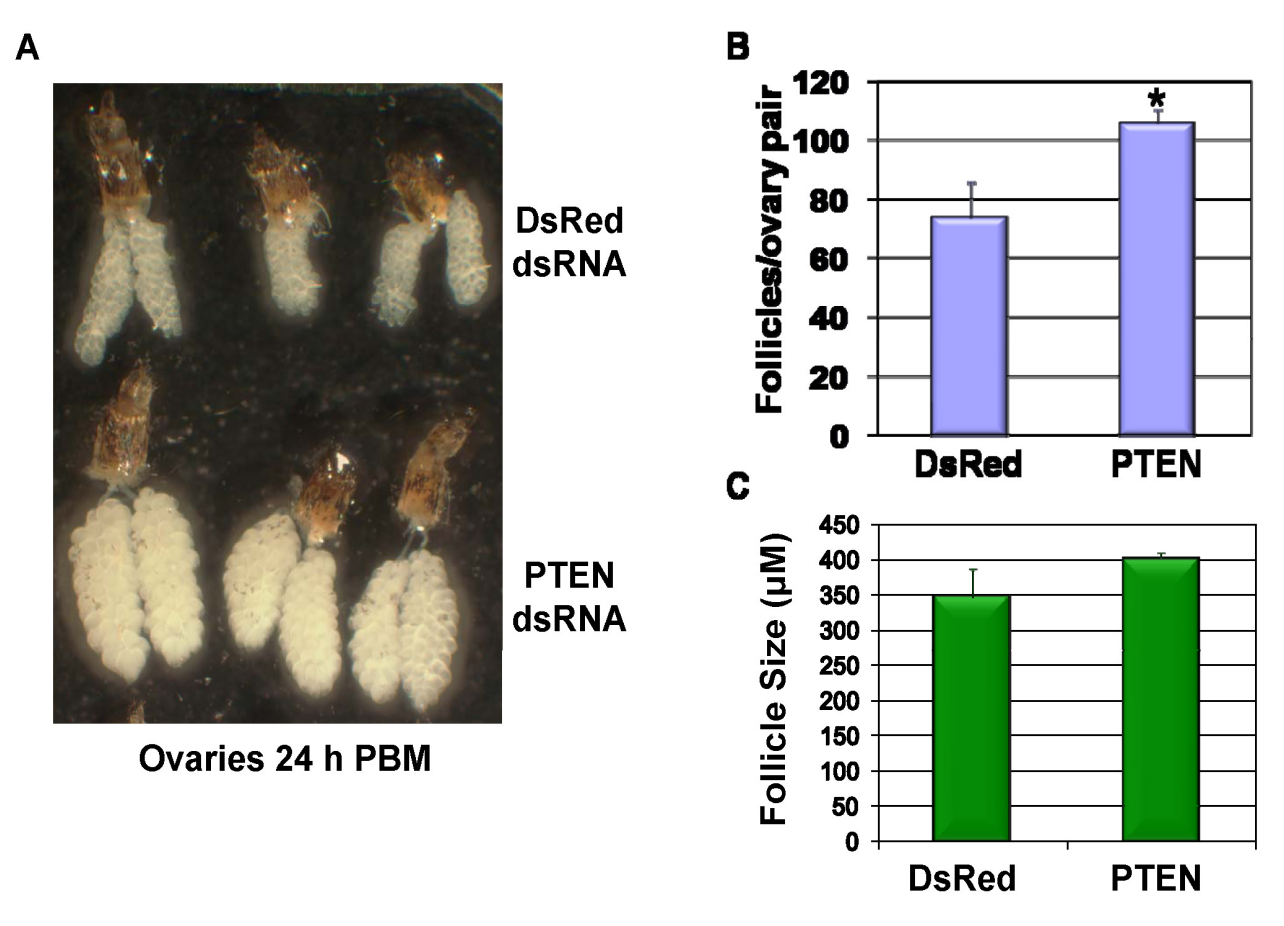
**The effect of AaegPTEN knockdown on egg development**: Newly eclosed *Ae. aegypti *were injected with 1.4 μg of dsRNA and allowed to recover for 48 h prior to blood feeding. **A**. Representative ovaries (24 h post bloodmeal) dissected from mosquitoes injected with dsRNA against AaegPTEN or DsRed (Control). **B**. Mosquitoes inoculated with AaegPTEN dsRNA produced significantly (* p = 0.0028) more mature follicles (106/ovary pair; n = 17) than the DsRed controls (74/ovary pair; n = 9). There was no significant difference in average follicle size (p = 0.31).

In the abdomen/fat body, we achieved 90% and 98% knockdown of AaegPTEN6 transcript with AaegPTEN and AaegPTEN6 dsRNA injections respectively (Figure 4A). This was significantly less than the DsRed (AaegPTEN vs. DsRed p = 0.02; AaegPTEN6 vs. DsRed p = 0.002) or AaegPTEN3 (AaegPTEN vs. AaegPTEN3 p = 0.014; AaegPTEN6 vs. AaegPTEN3 p = 0.002) controls. In the ovaries, we achieved a 51% and 99% knockdown of AaegPTEN6 transcript after injections with AaegPTEN and AaegPTEN6 dsRNA respectively (Figure 4B). Due to our small sample size (three qRT-PCR experiments; ten pooled ovaries/experiment; samples run in duplicate) these results were not significant (p = 0.081), however the trend was consistent with the fat body results. At both 24 and 48 h post bloodmeal, the ovaries of females injected with universal AaegPTEN dsRNA had the same number of, but more developed follicles (Figure 5) compared to the DsRed controls (p = 0.0028).

**Figure 6:**
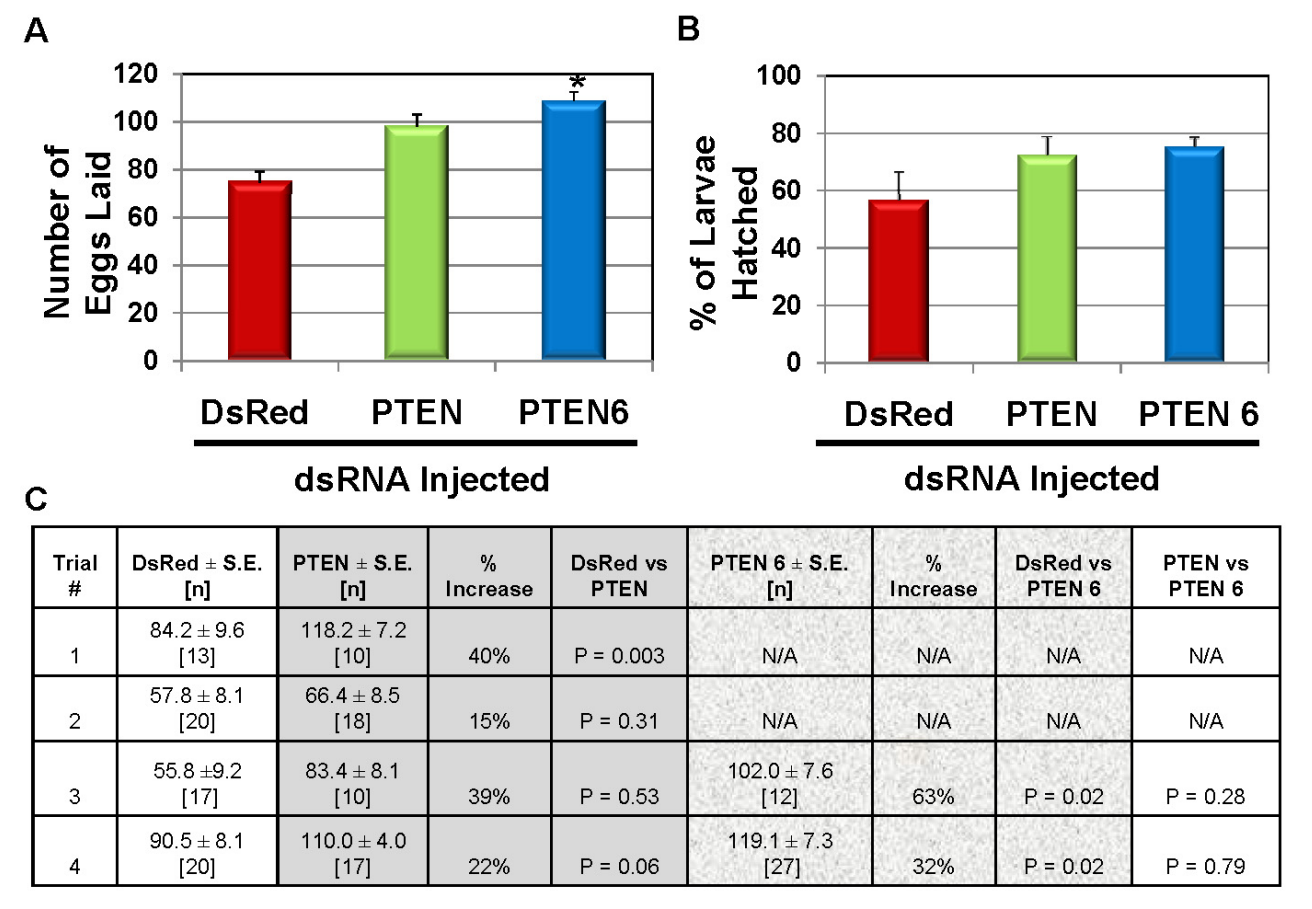
**Effects of RNAi-mediated knockdown of AaegPTEN and PTEN 6 on egg production and viability**: **A**. Female mosquitoes injected with AaegPTEN dsRNA laid significantly more eggs than control females injected with DsRed dsRNA. AaegPTEN6 dsRNA injected females also laid significantly higher number of eggs than DsRed control females. **B**. There was no significant difference in the viability of eggs laid by AaegPTEN or AaegPTEN6 dsRNA injected females and DsRed control females. **C**. Data from 4 different trials of RNAi Experiments. Solid highlights represent DsRed controls compared to AaegPTEN. Textured highlights represent the DsRed control compared to AaegPTEN6. Mean number of eggs and % larvae hatched from different trials is shown in the bar graphs. Bars followed by different letter are significantly different. The error bars represent standard error. The asterisk represents significant difference from the control group.

Female mosquitoes injected with dsRNA specifically targeting the AaegPTEN6 splice variant laid an average of 51% more eggs than the DsRed control females (p = 0.045). Female mosquitoes injected with the universal AaegPTEN dsRNA also laid an average of 31% more eggs than control mosquitoes injected with DsRed dsRNA (Figure 6a; p = 0.06). The number of eggs laid by the mosquitoes injected with AaegPTEN and AaegPTEN6 did not differ significantly (p = 0.79). Egg viability was measured by hatching the eggs oviposited by each individual dsRNA injected female, counting the 2^nd ^instar larvae, and comparing that to the total number of eggs laid. The percentage of hatched larvae from the eggs laid by the AaegPTEN and AaegPTEN6 injected females was greater than DsRed dsRNA injected females, but did not differ significantly (p = 0.09). Importantly, this demonstrates that there was no decrease in egg fitness from mosquitoes producing artificially high numbers of eggs. The data from replicate trials are detailed in Figure 6c. Thus, knockdown of both AaegPTEN and AaegPTEN6 impacted the reproductive success of females by increasing egg production, while the viability of these eggs was unchanged or even increased slightly.

Since AaegPTEN3 protein was predominantly expressed in the head, we hypothesized that knockdown of AaegPTEN3 would have little or no effect on egg production or viability. To examine this hypothesis, dsRNA against the unique 3' terminus of AaegPTEN3 was injected in newly eclosed females under the same conditions as described above. In heads, we achieved a 24 and 40% knockdown of AaegPTEN3 transcript and in the midgut, we achieved a 61 and 67% knockdown after injections with AaegPTEN and AaegPTEN3 dsRNA injections. However, due to small sample sizes this decrease did not differ significantly (data not shown). We found no significant difference in the number or viability of eggs laid by females injected with AaegPTEN3 dsRNA compared to eggs laid by DsRed control females. However, this may be due to insufficient knockdown of the AaegPTEN3 transcript.

## Discussion

Egg production in the mosquito is a carefully orchestrated series of hormonal and cell signaling events and is regulated by two major physiological processes; steroidogenesis in the ovaries and vitellogenesis in the fat body. One of the key signaling cascades regulating both steroidogenesis and vitellogenesis is the IIS cascade. PTEN, being an inhibitor of this cascade, is thus an important regulator of egg production at the molecular level. RNAi-mediated knockdown of AaegPTEN and more specifically the AaegPTEN6 splice variant significantly increased the reproductive success of female mosquitoes during their first reproductive cycle. There are at least two mechanisms by which knockdown of PTEN expression could increase reproductive output. The most likely method is that increased IIS signaling results in increased ecdysteroid production in the ovary, increased yolk protein production in the fat body, or both. It has been demonstrated that insulin stimulates ecdysteroid production by the ovaries of *Ae. aegypti *that is regulated by the PI-3K/Akt pathway [8]. Increased IIS signaling in the ovary could lead to the secretion of additional 20-hydroxyecdysone, which in turn signals vitellogenesis in the fat body [28]. Alternatively, increased insulin signaling in the fat body may directly impact vitellogenesis. It was demonstrated that insulin, in addition to 20-HE, was required for full expression of vitellogenin in the fat body [15]. Since we observed efficient knockdown of AaegPTEN6 in both the fat body (98%) and the ovary (99%) it is possible that both of these physiological processes played a role in the increased egg production and viability that we observed.

A second possibility is that increased insulin signaling impairs the mosquitoes' ability to resorb eggs that are partially developed. Between 20 – 25 h post bloodmeal, a majority of follicles have some yolk deposits. Follicles that are insufficiently developed at that time get degraded and resorbed. In our study, ovaries dissected 24 h post bloodmeal from mosquitoes previously injected with AaegPTEN dsRNA had more developed follicles, which could be a result of over stimulation of vitellogenesis in the fat body. Increased production of vitellogenin might cause faster deposition of yolk in the follicles and hence, prevent them from degrading. A considerable percentage of vitellogenic follicles also get resorbed between 25–30 h post bloodmeal. However, ovary dissections at 48 h post bloodmeal showed a greater number of more developed follicles suggesting that fewer of the vitellogenic follicles were being resorbed.

A third possibility is that knockdown of AaegPTEN and increased insulin signaling impact the number of potential follicles in each ovary. In *Ae. aegypti *there is an average of 50 – 60 follicles in each ovary, although this number varies greatly [29]. This means that on average a maximum of 100 – 120 eggs can be produced in a single reproductive cycle. Our dsRNA injections were performed shortly after adult emergence when the ovaries are still being developed and prepared for reproduction. Insulin signaling has been shown to play an important role in growth, including body size, cell size and cell number [30]. Thus, it is possible that mosquitoes with AaegPTEN knockdown may have unchecked ovary growth and an increase in the number of ovarioles. We are currently exploring this possibility.

Egg viability after dsRNA injections was examined by measuring the percentage of larvae hatched. Since females have limited resources, we suspected that we might observe a decrease in egg viability due to a compromise in resource allocation towards individual egg provisions. Surprisingly, eggs laid by AaegPTEN and AaegPTEN6 dsRNA injected females were not only higher in number, but also had similar or even slightly increased rates of larval hatching compared to DsRed controls. The increased egg production and viability could be a factor of resource allocation favored towards reproduction by the female mosquitoes. After a bloodmeal, mosquitoes can accumulate energy reserves (lipids and glycogen) for survival and flight [31,32]. In mosquitoes with increased insulin signaling more of the bloodmeal resources may be devoted to egg production instead of energy reserves. Mosquitoes also partition and allocate the blood meal proteins between reproduction and energy storage, which can be impacted by factors such as larval nutrition [33], adult nutrition [34] and gonotrophic cycles [35]. For our experiments, the larval and adult nutrition of mosquitoes was controlled by rearing all mosquitoes under identical conditions. However, we examined the effects of dsRNA injections only for the first gonotrophic cycle. It would be interesting to analyze the effects of increased insulin signaling on subsequent gonotrophic cycles and adult survivorship. However, the transient nature of transcript knockdown with dsRNA injections would make lifelong studies impractical and necessitates the use of transgenic mosquitoes which we are actively pursuing.

Both splice variant AaegPTEN3 and 6 had different protein expression patterns in various tissues of adult female mosquitoes. AaegPTEN3 transcript is predominant in the head and midgut, whereas, the protein was strongly expressed in the head only. The abundance of AaegPTEN3 protein in the head is consistent with the transcript expression but the protein was only expressed at low levels in the midgut. AaegPTEN6 transcript is predominant in the ovary and fat body. However, the protein was expressed in all tissues examined. Invariable expression of AaegPTEN6 could be due to its higher stability as a protein since AaegPTEN6 transcript is expressed at low levels in all the tissues. During developmental stages, AaegPTEN6 transcript is strongly expressed in early embryos and also found at lower levels in all other immature stages. We found AaegPTEN6 protein in early embryos but not in larval stages. This supports the notion of Riehle and Brown [23] that AaegPTEN6 may play a role in early embryogenesis. During larval stages, there is a possibility that other AaegPTEN splice variants play a role in development.

The protein expression pattern of AaegPTEN6 in ovaries during a reproductive cycle closely resembles the expression patterns for other components of the IIS cascade such as the mosquito insulin receptor (MIR) [8] and AaegP110 [27]. The physiological events taking place in the ovary during a reproductive cycle correlate with the expression dynamics of the proteins involved in the IIS cascade. During the first 12 – 24 h post-bloodmeal, follicle cells surrounding the developing oocytes produce ecdysteroids that stimulates yolk protein synthesis in the fat body (vitellogenesis). After ~24 h the follicle cells switch roles from steroid production to eggshell synthesis. Insulin signaling is not involved in the eggshell formation and thus components of the IIS cascade, including AaegPTEN6, are not expressed during this time. However, after oviposition (~72 h post bloodmeal) the expression of AaegPTEN6 and other components of the IIS cascade is restored in the new primary follicle in preparation for next reproductive cycle [8,27].

The differential expression pattern of AaegPTEN3 and 6 raises the possibility of each splice variant affecting particular phenotypes. Although AaegPTEN6 protein is expressed in all tissues, its distinctive expression in the ovaries and fat body during a reproductive cycle suggests a significant effect on mosquito fecundity. Conversely, the AaegPTEN3 protein is predominantly present in the head and expressed uniformly during a reproductive cycle, suggesting a possible role in the IIS cascade's other physiological roles such as aging. The location of AaegPTEN3 inside the head is unknown. Inefficient knockdown of AaegPTEN3 in the head suggest that it is localized in the nervous tissue and not the pericerebral fatbody, since nervous tissue is more resistant to RNAi [36].

Identification of AaegPTEN6 as a direct regulator of egg production may enable us to directly target the reproductive success of mosquitoes. Reproduction is one consideration when determining the efficiency of vector-borne disease transmission [37]. Therefore, a direct target of mosquito reproduction could be an advantageous new tool to augment present control strategies. Since resistance to insecticides, the most frequently used weapon against vector-borne diseases is increasing, our findings present AaegPTEN6 as a promising target against a range of mosquito-borne diseases.

## Conclusion

Insulin signaling regulates a diverse range of physiological processes, including reproduction. It has been shown that disruption of the IIS cascade can, in some cases, lead to reduced egg production or sterility. However, increased reproduction due to enhanced insulin signaling has not been previously observed. In this paper we demonstrate that manipulation of the IIS cascade can result in a marked increase in egg production during the mosquito's first reproductive cycle. This was accomplished through the knockdown of an IIS inhibitor, AaegPTEN, in two key reproductive tissues, the ovary and fat body. Increases in egg production of up to 63% were observed when all AaegPTEN splice variants were disrupted or specifically the AaegPTEN6 splice variant.

## Methods

### 1. Mosquito Rearing

The *Aedes aegypti *mosquitoes were reared at 27°C and 75% relative humidity in a 16/8 h light/dark photoperiod. Larvae were fed a mixture of ground rabbit chow : lactalbumin : brewer yeast (1 : 1 : 1). Adults were provided with a 10% dextrose solution ad libitum via a wick. Females were fed on porcine blood with 0.038% sodium citrate added as a preservative. Blood feeding was performed using artificial membranes with membrane feeders attached to a circulating water bath maintained at 37°C.

### 2. Splice variant specific antibodies generation

We generated peptide-based polyclonal antibodies against the C-terminus of the AaegPTEN3 and 6 splice variants. Since only the C-terminal amino acids are different for each variant we had limited options for antibody selection. AaegPTEN3 has a 101 amino acid tail providing us with some flexibility. We selected an 18 amino acid peptide (VNSPAESLVRYRLLSEPE) predicted to be highly antigenic on the unique C-terminal tail of AaegPTEN3. In contrast, AaegPTEN6 has only six unique amino acids. Thus, we generated a ten amino acid peptide (GWDSGESTYL), including four amino acids (GWDS) common to all three variants. The peptides were conjugated to KLH and injected into two rabbits by Proteintech Group, Inc. Neat serum from both rabbits injected with AaegPTEN6 worked extremely well on mosquito tissue samples. The AaegPTEN3 serum recognized several non-specific bands on mosquito samples and was therefore affinity purified with nickel-chelating resin (Aminolink Plus, Pierce) and the peptide used to generate the AaegPTEN3 antibody. Affinity purification resulted in a marked increase in specificity.

### 3. Protein extraction and immunoblot analysis

Immunoblot analysis was performed to assess the protein expression pattern of AaegPTEN3 and 6. Protein extracts were prepared from early eggs (0–6 h post oviposition), late eggs (> 48 h post oviposition), 2^nd ^instar larvae, 4^th ^instar larvae, early pupae (0–4 h post pupation), late pupae (48 h post pupation) and 3–5 day old adult males and adult females. Adult females were further separated by tissue type into head, midgut, fat body/abdominal body wall and ovary samples. To assess protein expression during a reproductive cycle, female mosquitoes were bloodfed and tissue samples were collected at various time points (0 (non-fed), 3, 6, 12, 24, 36, 48, and 72 hr (post oviposition)) from fully engorged females. Tissues from 5–10 mosquitoes were collected into 50 ul of 10× Complete protease Inhibitor (PI; Roche Diagnostics) in Aedes saline (125 mM NaCl, 5 mM KCl, 1.85 mM CaCl2, pH adjusted to 6.5 using NaOH). Protein extraction was facilitated by homogenizing the samples with a pestle in 4× loading buffer, (200 mMTris (adjusted to pH 6.8), 10% SDS, 50% glycerol, 400 mM DTT, 0.1% Coomassie Blue, in deionized water). After tissue disruption, the samples were boiled for 10 minutes at 95°C and then chilled on ice for 5 minutes. One to two tissues equivalents were loaded on to a 12% SDS-Page gel (Pierce or ISC BioExpress) and size fractionated. The proteins were transferred to a PVDF membrane (Whatman) and blocked with 5% low-fat dried milk in TBS and 0.1% Tween20 (TBST) overnight at 4°C with gentle rocking. Primary antibodies were diluted in fresh blocking solution (AaegPTEN3 1:1000 dilution; AaegPTEN6 1:25,000 dilution) and incubated at room temperature (RT) for 2 h. The blot was subsequently washed 3× for 30 minute at RT with TBST followed by incubation with an anti-mouse-HRP secondary antibody (400 μg/ml dilution in TBST; Pierce, Rockford, IL) for 2 h at RT. The blot was again washed 3× with TBST for 30 minute at RT followed by chemiluminescent detection (Pierce Super-Signal dura) on HyBlot CL autoradiography film (Denville Scientific). As a loading control, primary antibody against glyceraldehyde 3-phosphate dehydrogenase (GAPDH) was used. GAPDH was diluted in 5% BSA in TBST (1:10,000 dilution) and incubated at RT for 2 h followed by subsequent steps as described above.

### 4. RNAi-mediated knockdown Experiments

Double stranded RNA (dsRNA) was generated against both AaegPTEN3 and 6 splice variants, as well as a "universal" AaegPTEN dsRNA that recognized all six splice variants (Figure 3). The AaegPTEN3 dsRNA was generated against ~300 bp of its unique 3' terminus of the coding region. AaegPTEN6 has only six unique amino acids, therefore, AaegPTEN6 dsRNA was generated against ~300 bp of the unique 18 bp coding sequence and the 3' UTR. The pGEM-T Easy vectors (Promega) containing AaegPTEN, AaegPTEN3 and 6 sequences were used as templates for dsRNA production. To facilitate *in vitro *transcription, PCR amplification was performed to attach a T7 promoter sequence (TAATACGACTCACTATAGGG) to the 5' and 3' end of the target sequences. The PCR product was purified (Invitrogen PCR cleanup kit) and used in combination with the RiboMAX Large Scale RNA Production System (Promega) to generate sense and anti-sense RNA strands that were subsequently annealed together to generate the final dsRNA product. As a control, a similarly sized dsRNA fragment was generated against the red fluorescent protein, DsRed. The dsRNAs were concentrated to 4 μg/μl using a speedVac.

Virgin female mosquitoes (48 h post eclosion) were injected with 2 μg of dsRNA (0.5 μl of a 4 μg/μl solution). The injected mosquitoes recovered for 48 h in a humid chamber, were mated with male mosquitoes and provided with a bloodmeal. At 24 h post bloodmeal, ovaries were dissected to examine egg development. At 48 h post bloodmeal we dissected ovaries, counted the number of developed follicles and measured the size of individual follicles with an ocular micrometer on a stereo microscope set at 40×. Also, at 48 h post bloodmeal we dissected tissues from a subset of the females to look at transcript knockdown relative to DsRed controls. To assess the transcript knockdown, total RNA (Qiagen) was extracted from the dissected tissues (head, abdomen, midgut and ovary) of ten dsRNA injected females. Total RNA (1 μg) was used to make cDNA (Applied Biosystems) that then served as template for qRT-PCR (ABI Real-Time PCR) using primers specific to AaegPTEN3, AaegPTEN6 and actin as a control (Riehle and Brown, 2007). The qRT-PCR experiments were replicated three times and each sample tested in duplicate. Differences in normalized gene expression levels were compared by Kruskal-Wallace test. Due to small sample sizes, multiple pairwise comparisons were conducted using the Conover-Inman method. After other sample collections at 48 hr post bloodmeal, 15–20 females were individually provided with an oviposition substrate to oviposit eggs. After a 3 day oviposition period, the eggs were counted and compared to the DsRed controls. To look at the affects of dsRNA injections on egg viability, the eggs were dried on paper for ~24 h, hatched and the total number of 2^nd ^instar larvae was counted. Because data did not conform to parametric assumptions, they were analyzed using non-parametric methods. For 2-group experiments (DsRed vs. PTEN3), data were analyzed by Mann-Whitney U test. For 3-group experiments (DsRed vs. PTEN vs. PTEN6), data were analyzed by Kruskal-Wallace test, using the Dwass-Steel-Critchlow-Fligner method for multiple pairwise comparisons. Variances among trials were not significant (squared ranks test, p > 0.05) and data were pooled for analysis.

## Authors' contributions

MAR and AJA designed and planned the experiments. AJA and KMQ performed the experiments. JLR performed the statistical analysis. AJA and MAR wrote the manuscript. All authors read and approved the final manuscript.

## References

[B1] Kyle JL, Harris E (2008). Global spread and persistence of dengue. Annu Rev Microbiol.

[B2] Holmes DJ, Fluckiger R, Austad SN (2001). Comparative biology of aging in birds: an update. Exp Gerontol.

[B3] Sgro CM, Partridge L (1999). A delayed wave of death from reproduction in Drosophila. Science.

[B4] Jenkins NL, McColl G, Lithgow GJ (2004). Fitness cost of extended lifespan in Caenorhabditis elegans. Proc Biol Sci.

[B5] Partridge L, Gems D, Withers DJ (2005). Sex and death: what is the connection?. Cell.

[B6] Furnari FB, Lin H, Huang HS, Cavenee WK (1997). Growth suppression of glioma cells by PTEN requires a functional phosphatase catalytic domain. Proc Natl Acad Sci USA.

[B7] Myers MG, Mendez R, Shi P, Pierce JH, Rhoads R, White MF (1998). The COOH-terminal tyrosine phosphorylation sites on IRS-1 bind SHP-2 and negatively regulate insulin signaling. J Biol Chem.

[B8] Riehle MA, Brown MR (1999). Insulin stimulates ecdysteroid production through a conserved signaling cascade in the mosquito Aedes aegypti. Insect Biochem Mol Biol.

[B9] Hsin H, Kenyon C (1999). Signals from the reproductive system regulate the lifespan of C. elegans. Nature.

[B10] Kenyon C (2001). A conserved regulatory system for aging. Cell.

[B11] Clancy DJ, Gems D, Harshman LG, Oldham S, Stocker H, Hafen E, Leevers SJ, Partridge L (2001). Extension of life-span by loss of CHICO, a Drosophila insulin receptor substrate protein. Science.

[B12] Flatt T, Min KJ, D'Alterio C, Villa-Cuesta E, Cumbers J, Lehmann R, Jones DL, Tatar M (2008). Drosophila germ-line modulation of insulin signaling and lifespan. Proc Natl Acad Sci USA.

[B13] Riehle MA, Brown MR (2002). Insulin receptor expression during development and a reproductive cycle in the ovary of the mosquito Aedes aegypti. Cell Tissue Res.

[B14] Kang MA, Mott TM, Tapley EC, Lewis EE, Luckhart S (2008). Insulin regulates aging and oxidative stress in Anopheles stephensi. J Exp Biol.

[B15] Roy SG, Hansen IA, Raikhel AS (2007). Effect of insulin and 20-hydroxyecdysone in the fat body of the yellow fever mosquito, Aedes aegypti. Insect Biochem Mol Biol.

[B16] Vazquez F, Grossman SR, Takahashi Y, Rokas MV, Nakamura N, Sellers WR (2001). Phosphorylation of the PTEN tail acts as an inhibitory switch by preventing its recruitment into a protein complex. J Biol Chem.

[B17] Tang X, Powelka AM, Soriano NA, Czech MP, Guilherme A (2005). PTEN, but not SHIP2, suppresses insulin signaling through the phosphatidylinositol 3-kinase/Akt pathway in 3T3-L1 adipocytes. J Biol Chem.

[B18] Reddy P, Liu L, Adhikari D, Jagarlamudi K, Rajareddy S, Shen Y, Du C, Tang W, Hamalainen T, Peng SL, Lan ZJ, Cooney AJ, Huhtaniemi I, Liu K (2008). Oocyte-specific deletion of Pten causes premature activation of the primordial follicle pool. Science.

[B19] Fan HY, Liu Z, Cahill N, Richards JS (2008). Targeted disruption of Pten in ovarian granulosa cells enhances ovulation and extends the life span of luteal cells. Mol Endocrinol.

[B20] Cavaliere V, Donati A, Hsouna A, Hsu T, Gargiulo G (2005). dAkt kinase controls follicle cell size during Drosophila oogenesis. Dev Dyn.

[B21] Sarquis MS, Agrawal S, Shen L, Pilarski R, Zhou XP, Eng C (2006). Distinct expression profiles for PTEN transcript and its splice variants in Cowden syndrome and Bannayan-Riley-Ruvalcaba syndrome. Am J Hum Genet.

[B22] Maehama T, Kosaka N, Okahara F, Takeuchi K, Umeda M, Dixon JE, Kanaho Y (2004). Suppression of a phosphatidylinositol 3-kinase signal by a specific spliced variant of Drosophila PTEN. FEBS Lett.

[B23] Riehle MA, Brown JM (2007). Characterization of Phosphatase and Tensin Homolog expression in the mosquito Aedes aegypti: Six splice variants with developmental and tissue specificity. Insect Mol Biol.

[B24] Hwangbo DS, Gershman B, Tu MP, Palmer M, Tatar M (2004). Drosophila dFOXO controls lifespan and regulates insulin signalling in brain and fat body. Nature.

[B25] Wolkow CA, Kimura KD, Lee MS, Ruvkun G (2000). Regulation of C. elegans life-span by insulinlike signaling in the nervous system. Science.

[B26] Murphy CT, Lee SJ, Kenyon C (2007). Tissue entrainment by feedback regulation of insulin gene expression in the endoderm of Caenorhabditis elegans. Proc Natl Acad Sci USA.

[B27] Pri-Tal BM, Brown JM, Riehle MA (2008). Identification and characterization of the catalytic subunit of phosphatidylinositol 3-kinase in the yellow fever mosquito Aedes aegypti. Insect Biochem Mol Biol.

[B28] Raikhel AS, Kokoza VA, Zhu J, Martin D, Wang SF, Li C, Sun G, Ahmed A, Dittmer N, Attardo G (2002). Molecular biology of mosquito vitellogenesis: from basic studies to genetic engineering of antipathogen immunity. Insect Biochem Mol Biol.

[B29] Clements AN, Boocock MR (1984). Ovarian development in mosquitoes: stages of growth and arrest, and follicular resorption. Physiological Entomology.

[B30] Garofalo RS (2002). Genetic analysis of insulin signaling in Drosophila. Trends Endocrinol Metab.

[B31] Nayar JK, Sauerman DM (1975). The effects of nutrition on survival and fecundity in Florida mosquitoes. Part 1. Utilization of sugar for survival. J Med Entomol.

[B32] Clements AN, Clements AN (1992). The biology of mosquitoes.

[B33] Brazil DP, Hemmings BA (2001). Ten years of protein kinase B signalling: a hard Akt to follow. Trends Biochem Sci.

[B34] Gary RE, Foster WA (2001). Effects of available sugar on the reproductive fitness and vectorial capacity of the malaria vector Anopheles gambiae (Diptera: Culicidae). J Med Entomol.

[B35] Briegel H, Hefti M, DiMarco E (2002). Lipid metabolism during sequential gonotrophic cycles in large and small female Aedes aegypti. J Insect Physiol.

[B36] Buckingham SD, Esmaeili B, Wood M, Sattelle DB (2004). RNA interference: from model organisms towards therapy for neural and neuromuscular disorders. Hum Mol Genet.

[B37] Macdonald G (1957). The Epidemiology and Control of Malaria.

